# Supplementary material for: In Silico Screening of Metal−Organic Frameworks and Zeolites for He/N2 Separation
Source: Molecules. 2022 Dec 20;28(1):20. doi: 10.3390/molecules28010020 (PMC9822448; doi:10.3390/molecules28010020)
Supplement: Supplementary file 1 [file molecules-28-00020-s001.zip › molecules-2093453-supplementary.pdf]

Supplementary Materials for

In silico screening of metal–organic frameworks and zeolites for He/N_2 separation

Ivan V. Grenev[1,2,*], Vladimir Yu Gavrilov[2]

(1) Novosibirsk State University, Pirogova str. 1, Novosibirsk 630090, Russia

(2) Boreskov Institute of Catalysis, Ac. Lavrentiev av. 5, Novosibirsk 630090, Russia

* - author for correspondence, greneviv@catalysis.ru

Contents:

Section S1. Comparison of molecular simulations and experimental data 2

Section S2. Structure – adsorption performance relationships 5

Section S3. Comparison of ideal and mixture adsorption selectivities 8

Section S4. Relationship between API and APS metrics 9

Section S5. Comparison of the structural properties PDF for the best 50 MOF structures for VSA and PSA with all MOF structures...10

Section S6. Characteristics of Top 10 MOFs for adsorption-based separation 12

Section S7. Effect of electrostatic interaction on He/N_2 separation..13

Section S8. Structure – diffusion performance relationships...15

Section S9. Comparison of the structural properties PDF for the best 50 MOF structures for membrane separation process with all MOF structures. 17

Section S10. Chemical composition – membrane separation performance relationships.........18

Section S11. Characteristics of Top 10 MOFs for membrane-based separation. 19

References...20

Section S1. Comparison of molecular simulations and experimental data

Table S1. Comparison of adsorption capacities, adsorption selectivity and APS of zeolites 5A,13X and 4A for He and N_2 and CH4 at 100 1 bar and room temperature.

Zeolite name	N_{He}**(1 bar) [mol/kg]** [1]	N_{N_2}**(1 bar) [mol/kg]** [2]	$S_{ads,N_2/He}^{single}$	ΔN_{N2} **(VSA) [mol/kg]**	**APS [mol/kg]**
Zeolite 5A	1.17×10^{-3}	0.536	458	0.439	201
Zeolite 13X	1.31×10^{-3}	0.290	221	0.261	58
Zeolite 4A	5.75×10^{-3}	0.313	54	0.275	15

Table S2. Experimental N_2 adsorption data for the MOFs used in Figure 1 (left).

CCDC Identifier	MOF name	Pressure range [bar]	Temperature [K]	Reference
FIQCEN	Cu-BTC / HKUST-1	P < 1	298	[3]
FIQCEN	Cu-BTC / HKUST-1	1 < P < 8	295	[4]
EDUSIF	IRMOF-1	P < 1	298	[5]
EDUSIF	IRMOF-1	1 < P < 20	297	[6]
YUVSUE	Bio-MOF-11	P < 1	298	[7]
BEYSEF	Bio-MOF-12	P < 1	298	[7]
RUBTAK	UIO-66	P < 20	298	[8]
UNOBOR	Co3(btdc)3(bpy)2	P < 1	298	[9]

Table S3. Experimental N_2 adsorption data for the zeolites used in Figure 1 (right).

IZA Identifier	Zeolite name	Pressure range [bar]	Temperature [K]	Reference
BEA	Pure silica zeolite beta	P < 1	303	[10]
CHA	Pure silica chabazite	P < 1	303	[10]
FER	Siliceous ferrierite	P < 1	303	[10]
MFI	silicalite-1	P < 1	303	[10]
STT	SSZ-23	P < 1	303	[10]
AEL	AlPO-11	P < 1	296	[11]
ERI	AlPO-17	P < 1	298	[12]
AEI	AlPO-18	P < 1	298	[12]
ATT	AlPO-33	P < 1	298	[12]
ZON	UiO-7	P < 1	298	[12]

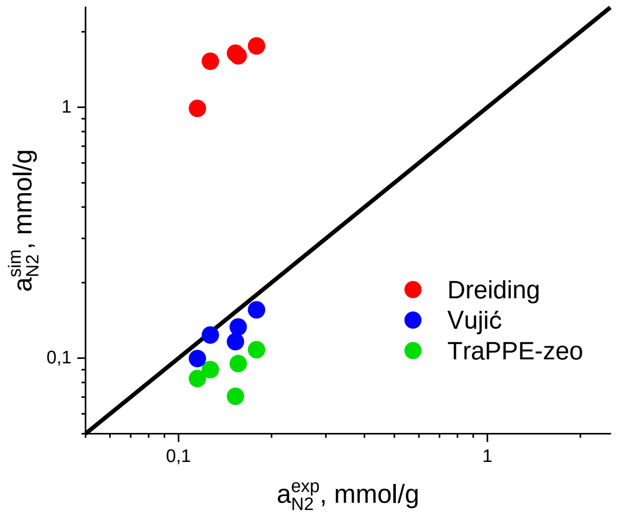

Figure S1. Comparison of calculated N_2 uptake at 303 K and 1 bar obtained using different force field models for 5 different pure silica structures (BEA, CHA, MFI, FER, STT) with the corresponding experimental data [10].

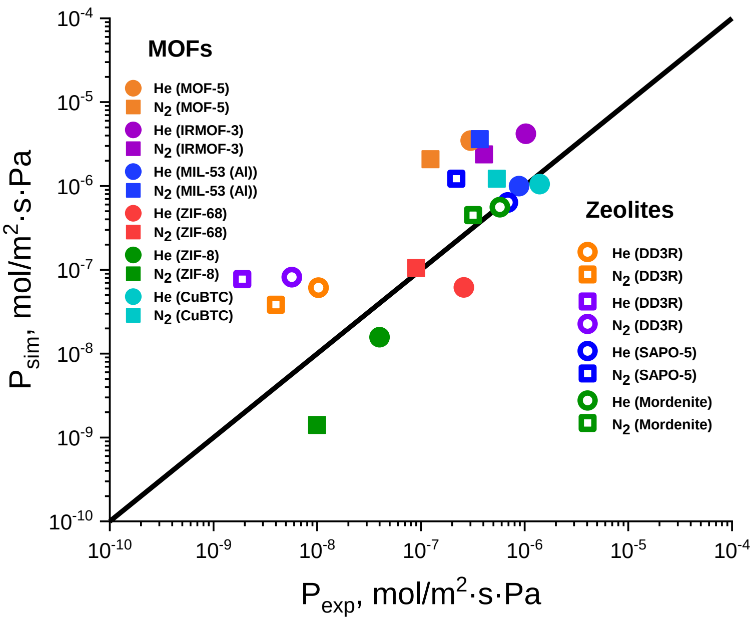

Figure S2. Comparison of experimental and molecular simulations data for He and N_2 permeance for MOFs and zeolites.

Table S4. Experimental He and N_2 permeance data for the MOFs used in Figure S2.

CCDC Identifier	MOF name	Molecule	Thickness [µm]	Pressure feed [bar]	Pressure permeate [bar]	Temperature [K]	Reference
FIQCEN	CuBTC	He (Single)	30	2	1	298	[13]
FIQCEN	CuBTC	N2 (Single)	30	2	1	298	[13]
EDUSIF	MOF-5	He (Single)	14	2.7	1.01	298	[14]
EDUSIF	MOF-5	N2 (Single)	14	2.7	1.01	298	[14]
QONQEQ	MIL-53 (Al)	He (Single)	60	0.6	Vacuum	298	[15]
QONQEQ	MIL-53 (Al)	N2 (Single)	60	0.6	Vacuum	298	[15]
GITTUZ	ZIF-68	He (Single)	40	2	1	298	[16]
GITTUZ	ZIF-68	N2 (Single)	40	2	1	298	[16]
OFERUN	ZIF-8	He (Single)	80	2	1	298	[17]
OFERUN	ZIF-8	N2 (Single)	80	2	1	298	[17]
EDUSUR	IRMOF-3	He (Single)	10	1	Vacuum	298	[18]
EDUSUR	IRMOF-3	N2 (Single)	10	1	Vacuum	298	[18]

Table S5. Experimental He and N_2 permeance data for the zeolites used in Figure S2.

IZA Identifier	Zeolite name	Molecule	Thickness [µm]	Pressure feed [bar]	Pressure permeate [bar]	Temperature [K]	Reference
DDR	DD3R	He (Single)	10	5	1	301	[19]
DDR	DD3R	N2 (Single)	10	5	1	301	[19]
AFI	SAPO-5	He (Single)	10	2	1	298	[20]
AFI	SAPO-5	N2 (Single)	10	2	1	298	[20]
MOR	Mordenite	He (Single)	20	2	1	298	[21]
MOR	Mordenite	N2 (Single)	20	2	1	298	[21]

Section S2. Structure – adsorption performance relationships

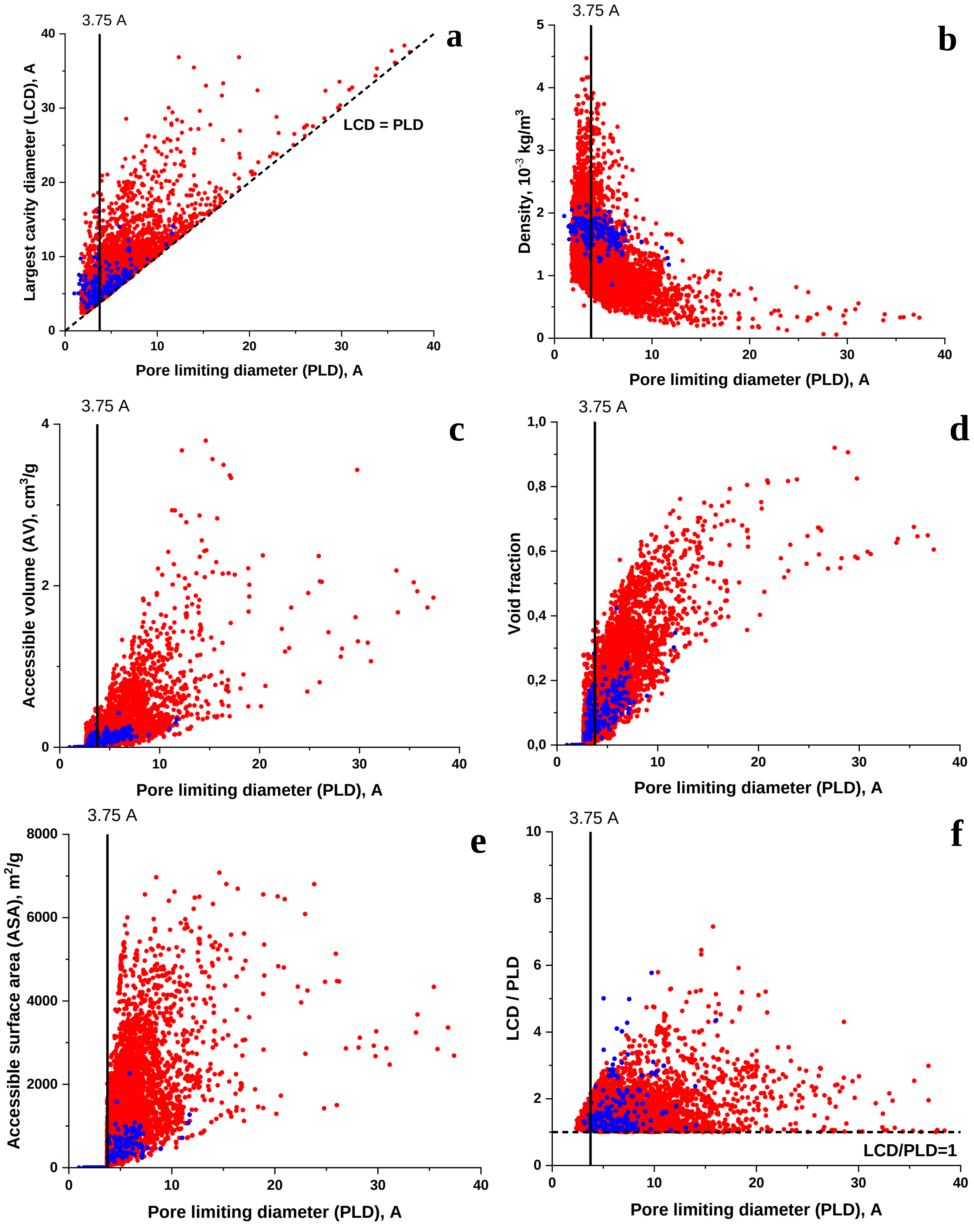

Figure S3. Comparison of the structural parameters of the initial MOF (red dots) and zeolites (blue dots) databases.

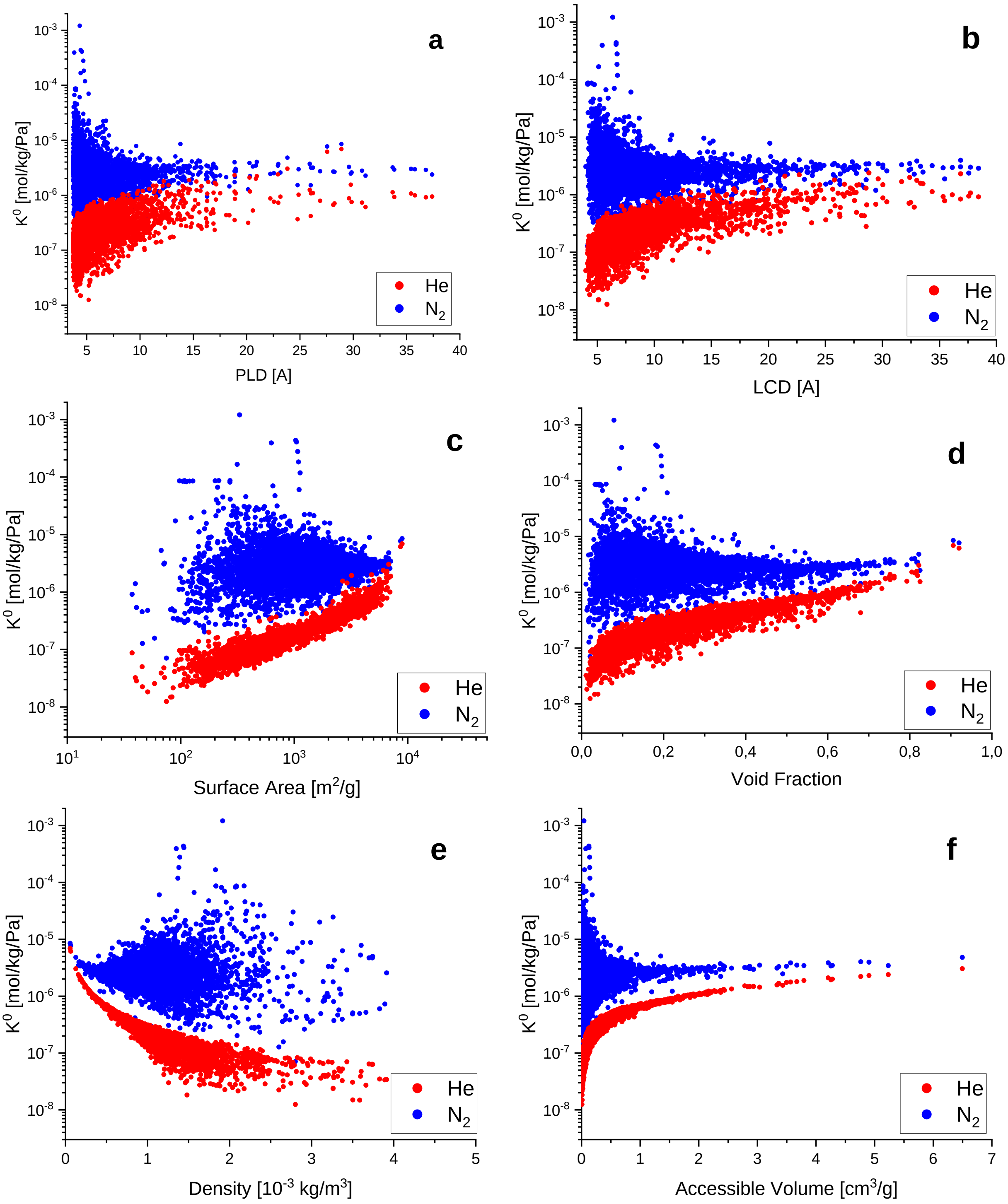

Figure S4. Henry's constant (K^0) versus pore limiting diameter (a), largest cavity diameter (b), accessible surface area (c), porosity (d), density (e) and accessible volume (f) for He and N_2.

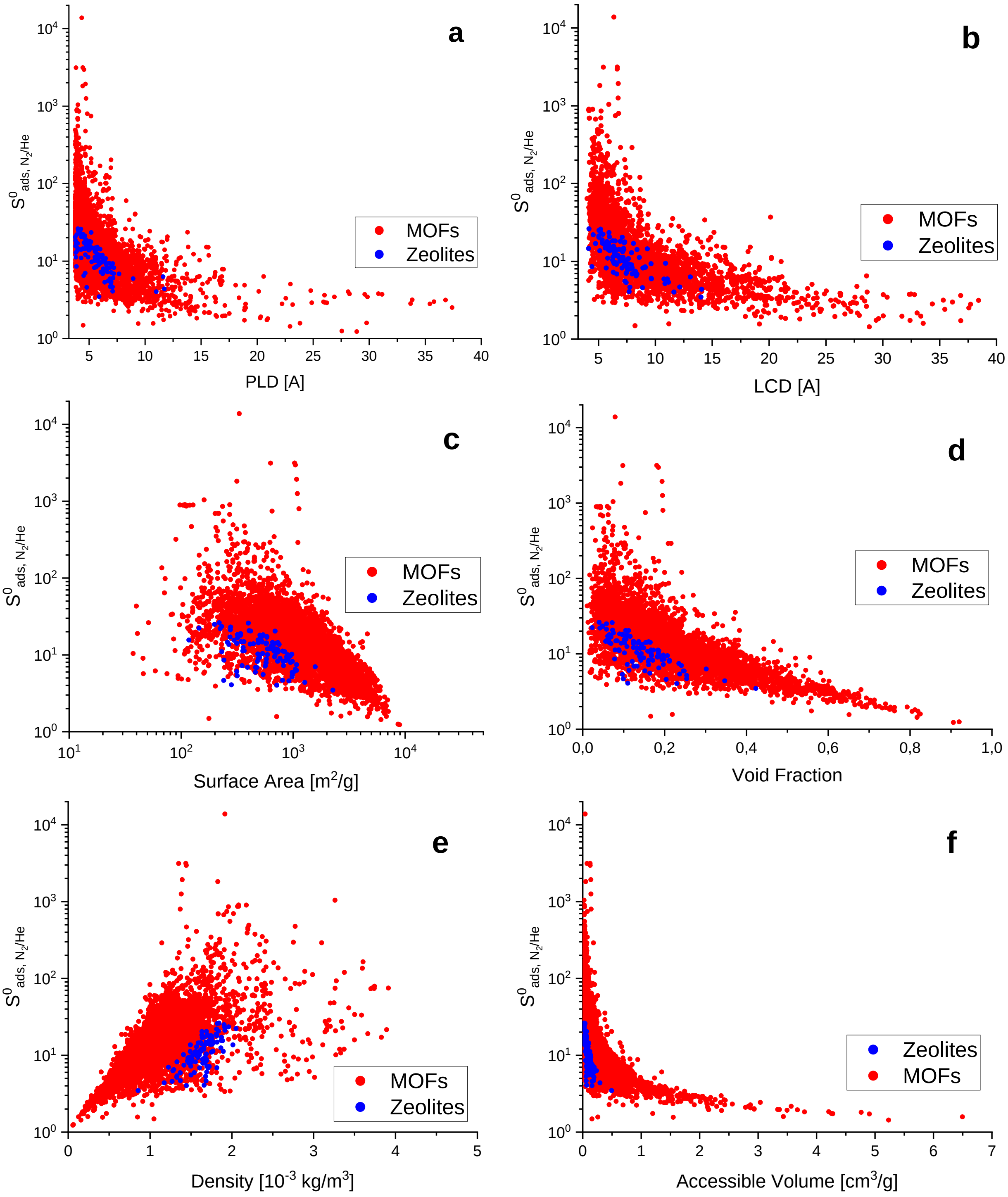

Figure S5. Adsorption selectivity ($S^{0}_{ads\ N_2/He}$) versus pore limiting diameter (a), largest cavity diameter (b), accessible surface area (c), porosity (d), density (e) and accessible volume (f) for MOF and zeolite structures.

Section S3. Comparison of ideal and mixture adsorption selectivities

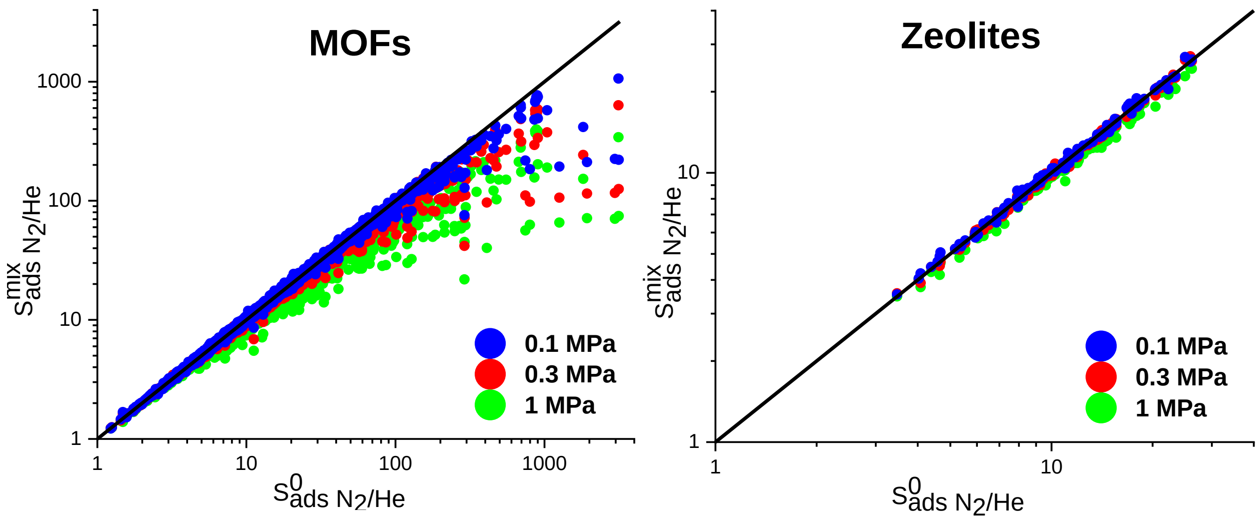

Figure S6. Comparison of ideal $S^{0}_{ads,N_2/He}$ and $S^{mix}_{ads,N_2/He}$ mixture adsorption selectivities of MOFs (left) and zeolites (right) at 1, 3, 10 bar. Bulk composition of the mixture is equimolar.

Section S4. Relationship between API and APS metrics

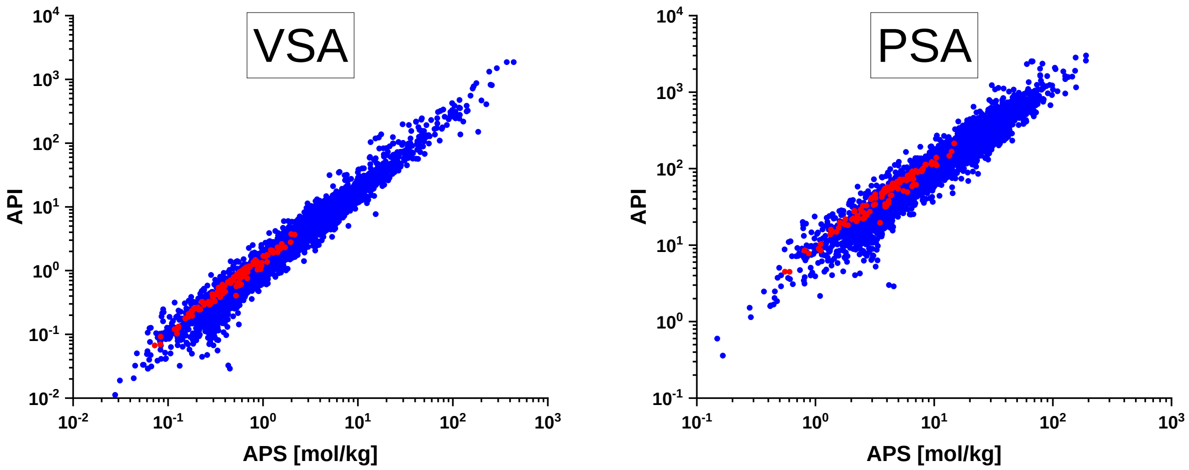

Figure S7. Relationship between API and APS metrics for vacuum swing adsorption (left) and pressure swing adsorption (right). Metal-organic frameworks are marked with blue dots, zeolites are marked with red dots.

Section S5. Comparison of the structural properties PDF for the best 50 MOF structures for VSA and PSA with all MOF structures

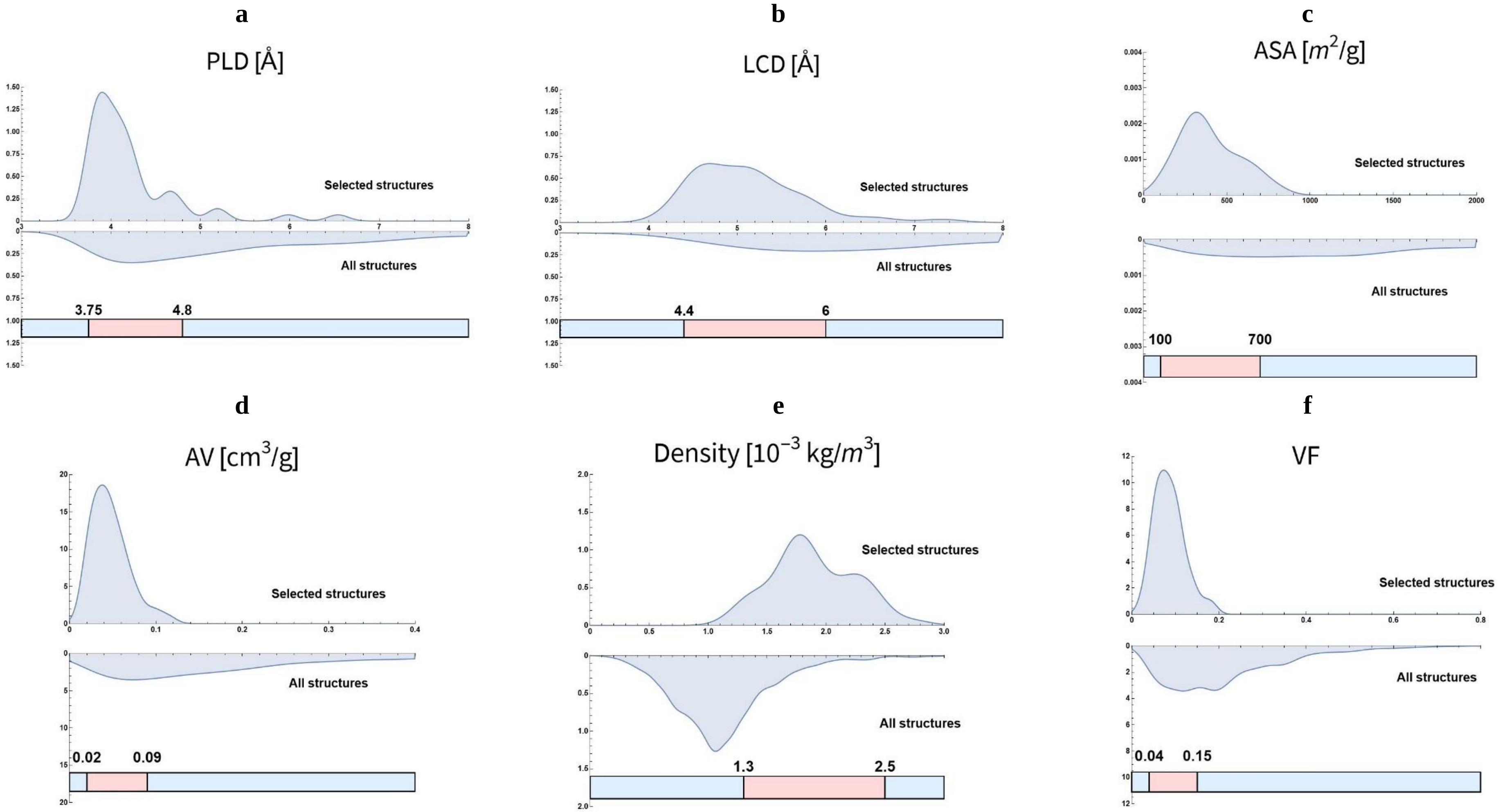

Figure S8. Comparison of the smoothed probability density distribution (PDF) of structural properties for the best 50 MOF structures with all MOF structures considered in this study for the VSA separation process. The bottom of each figure shows the range of effective structural properties that comprise over 90% of the Top 50 MOFs database for VSA.

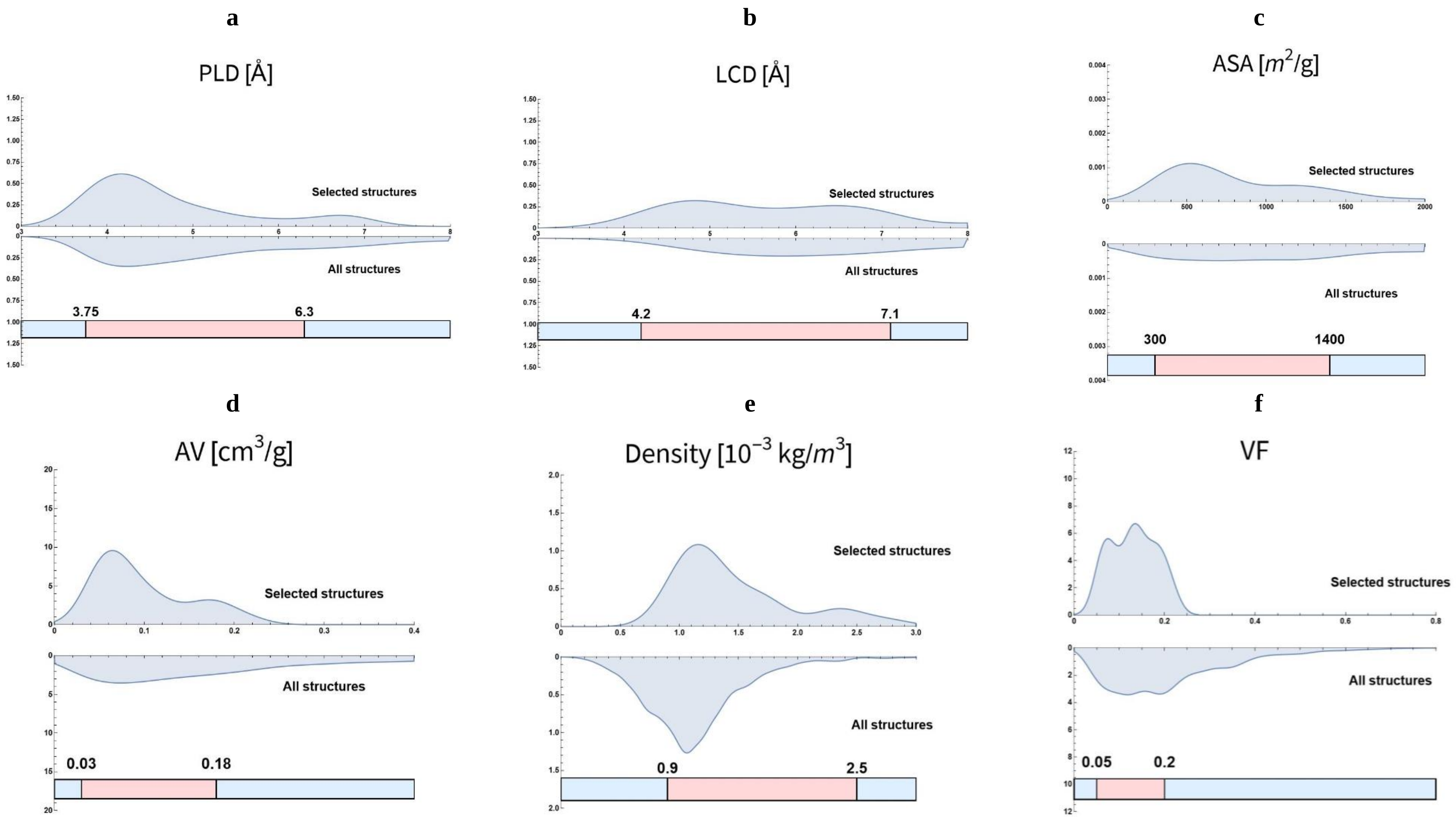

Figure S9. Comparison of the smoothed probability density distribution (PDF) of structural properties for the best 50 MOF structures with all MOF structures considered in this study for the PSA separation process. The bottom of each figure shows the range of effective structural properties that comprise over 90% of the Top 50 MOFs database for PSA.

Section S6. Characteristics of Top 10 MOFs for adsorption-based separation

Table S6. Characteristics of Top 10 MOFs for VSA separation.

Type	Structure	LCD [Å] / PLD [Å]	R	ΔN [mol/kg]	$S_{ads,N_2/He}^{mix}$	API	APS [mol/kg]
MOF	SEHVOQ	5.95 / 3.89	0.85	1.35	324.4	1858	438
MOF	FIVQOR	4.64 / 4.07	0.83	1.03	359.4	1861	370
MOF	ZUTBUN	4.44 / 3.87	0.83	0.91	320.9	1493	290
MOF	UZATAR	4.92 / 3.77	0.83	0.71	361.1	809	257
MOF	MITGOL	4.98 / 3.86	0.85	0.72	348.7	822	250
MOF	PONPOX	4.57 / 3.78	0.82	0.85	286.0	1322	242
MOF	POKVAO	5.22 / 4.00	0.80	0.56	400.9	406	225
MOF	WAZCUX	5.01 / 4.11	0.86	0.64	315.2	467	200
MOF	XUJSAY	5.28 / 3.82	0.82	0.43	428.4	150	185
MOF	MUVMOG	4.19 / 3.80	0.88	0.92	191.9	870	177

Table S7. Characteristics of Top 10 MOFs for PSA separation.

Type	Structure	LCD [Å] / PLD [Å]	R	ΔN [mol/kg]	$S_{ads,N_2/He}^{mix}$	APS [mol/kg]
MOF	POLBIB	4.83 / 4.17	0.83	2.41	79.1	190
MOF	CAYKEU	6.78 / 5.32	0.81	2.24	85.1	190
MOF	ZAGHAT	4.21 / 3.99	0.82	2.08	75.7	157
MOF	COQNIF	8.24 / 5.80	0.83	2.95	52.7	156
MOF	TOPKIT	8.18 / 5.07	0.83	3.49	44.1	154
MOF	NEYZAU	5.01 / 4.01	0.82	3.19	45.7	146
MOF	KIRTUC	4.52 / 3.79	0.81	2.14	63.3	135
MOF	ABULOB	4.51 / 3.77	0.82	2.10	60.8	127
MOF	UXABOL	5.11 / 4.47	0.82	1.32	96.2	127
MOF	NEFZOO	4.77 / 4.31	0.83	2.51	50.6	127

Section S7. Effect of electrostatic interaction on He/N_2 separation

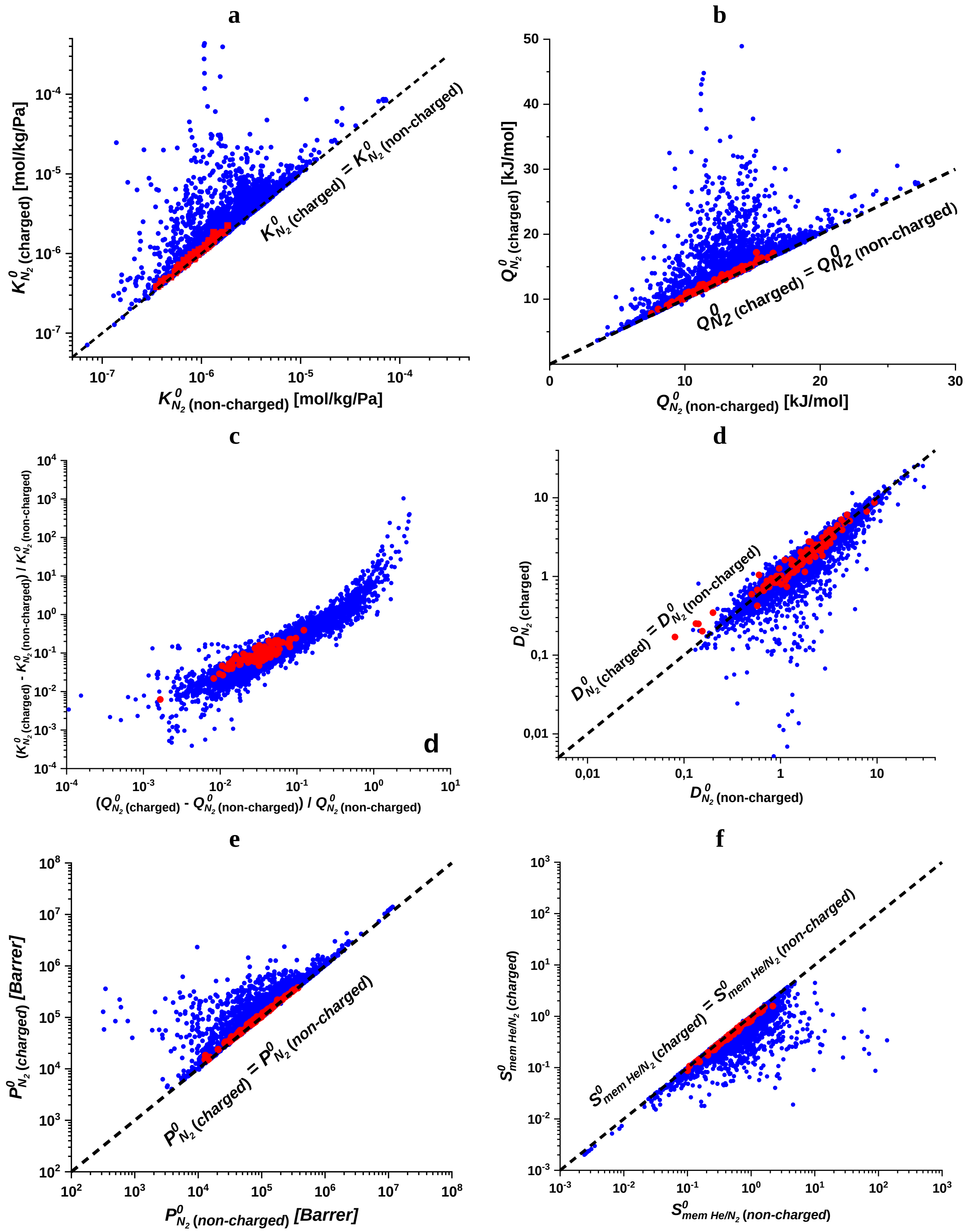

Figure S10. Influence of electrostatic interaction between N_2 molecules and adsorbent atoms on Henry's adsorption constant (a), adsorption enthalpy (b), Henry's constant - adsorption enthalpy relationship (c), diffusion coefficient (d), permeability (e) and membrane selectivity (f). Metal-organic frameworks are marked with blue dots, zeolites are marked with red dots.

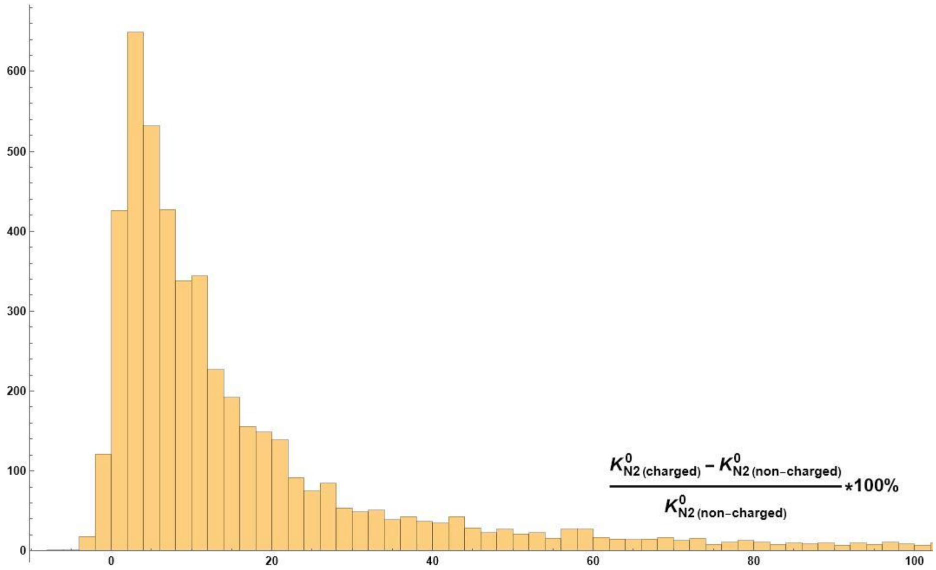

Figure S11. Influence of electrostatic interaction on N_2 Henry's adsorption constant.

Section S8. Structure – diffusion performance relationships

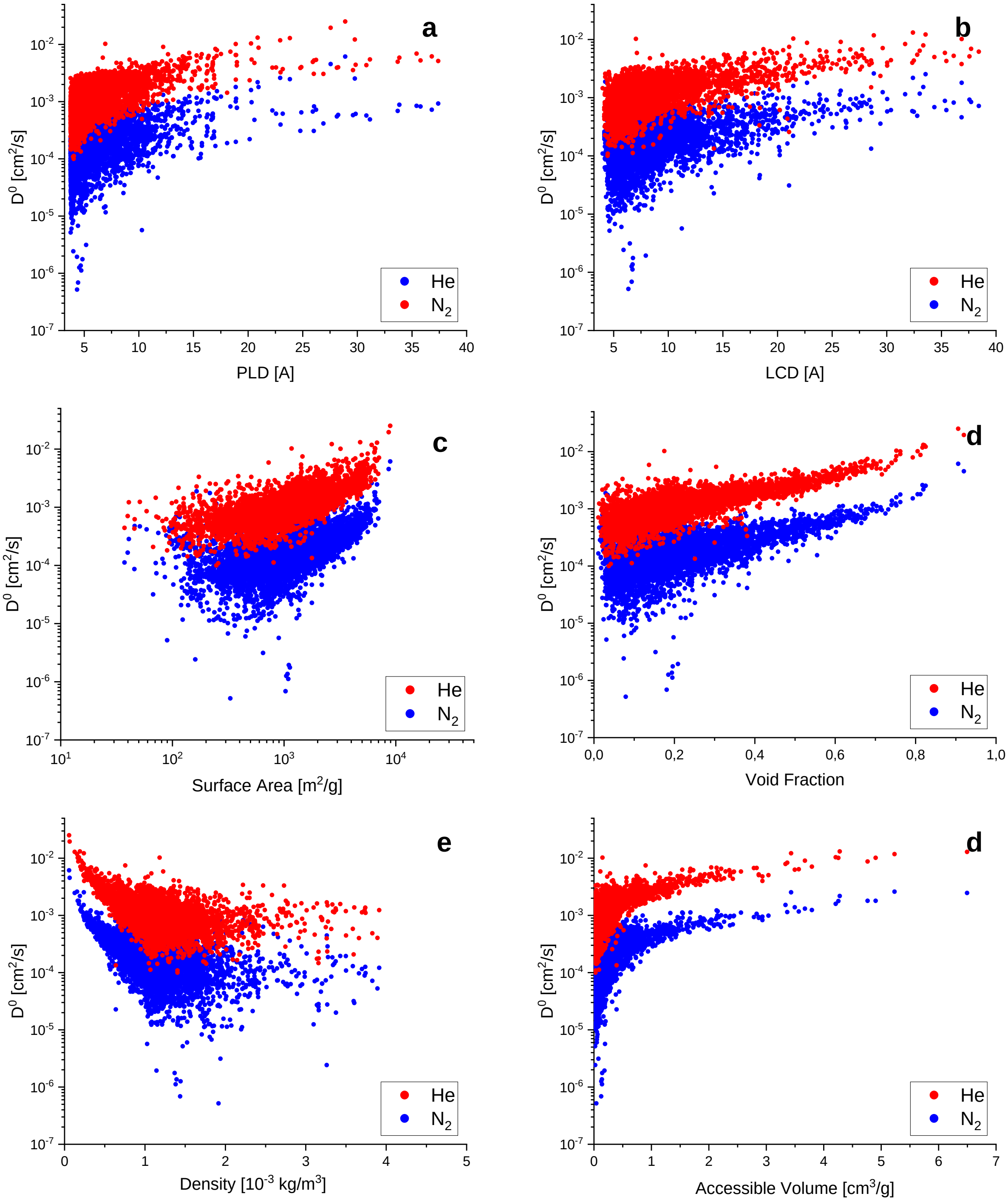

Figure S12. Diffusion coefficient (D^0) versus pore limiting diameter (a), largest cavity diameter (b), accessible surface area (c), porosity (d), density (e) and accessible volume (f) for He and N_2.

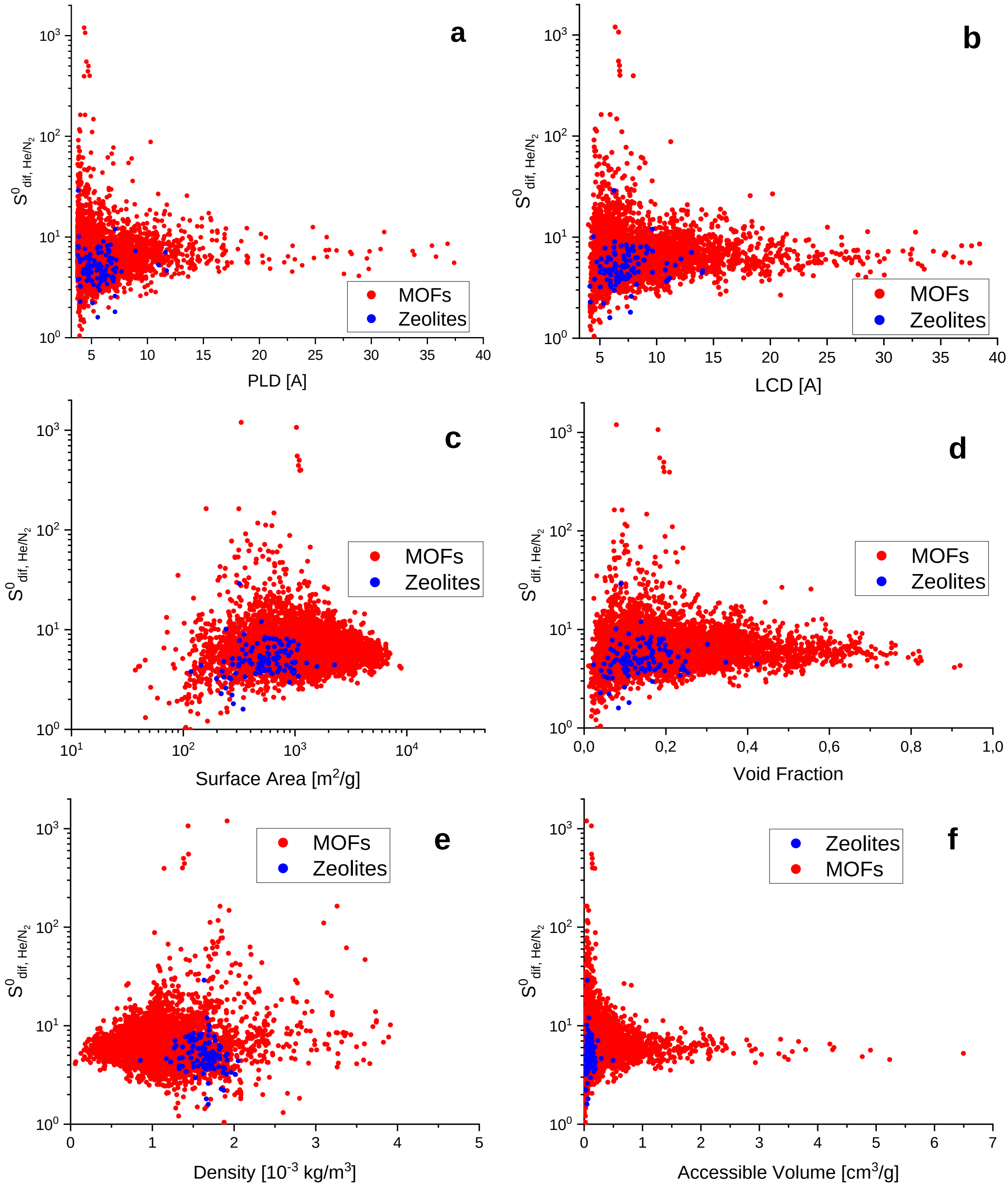

Figure S13. Diffusion selectivity ($S^0_{dif\ He/N_2}$) versus pore limiting diameter (a), largest cavity diameter (b), accessible surface area (c), porosity (d), density (e) and accessible volume (f) for MOF and zeolites structures.

Section S9. Comparison of the structural properties PDF for the best 50 MOF structures for membrane separation process with all MOF structures

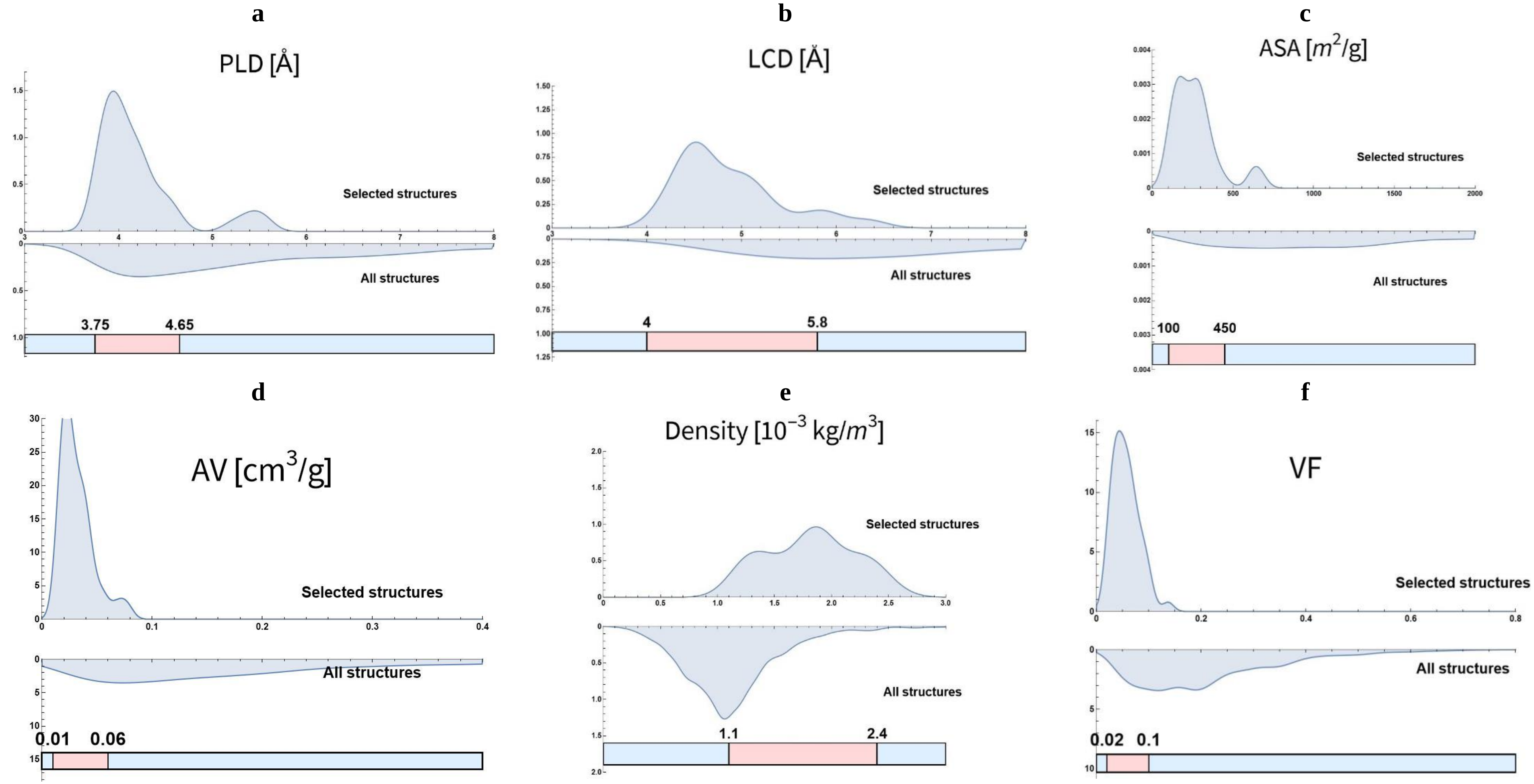

Figure S14. Comparison of the smoothed probability density distribution (PDF) of structural properties for the best 50 MOF structures with all MOF structures considered in this study for membrane separation process. The bottom of each figure shows the range of effective structural properties that comprise over 90% of the Top 50 MOF structures for membrane separation process.

Section S10. Chemical composition – membrane separation performance relationships

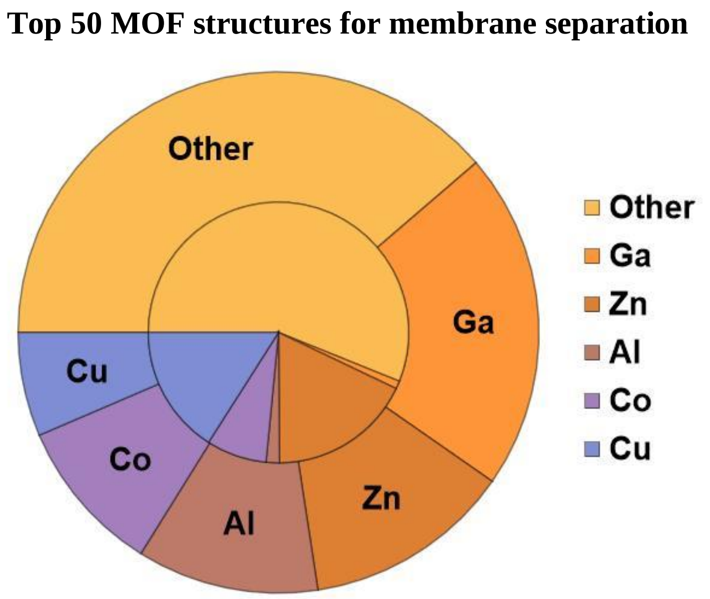

Figure S15. Chemical composition distribution of MOFs by metal type. The outer pie chart corresponds to the Top 50 best MOFs, the inner one corresponds to all MOFs considered in this study.

Section S11. Characteristics of Top 10 MOFs for membrane-based separation

Table S8. Characteristics of Top 10 MOFs for membrane separation.

CCDC Identifier	LCD [Å] / PLD [Å]	$S^{0}_{ads\ N_2/He}$	$S^{mix}_{ads\ N_2/He}$	$S^{0}_{dif\ He/N_2}$	$S^{mix}_{dif\ He/N_2}$	$P^{0}_{N_2}$[barrer]	$P^{mix}_{N_2}$[Barrer]	$S^{0}_{mem\ N_2/He}$	$S^{mix}_{mem\ N_2/He}$
MISQIQ *	4.16 / 3.92	885	565	2.1	1.3	1.22×10^{7}	3.20×10^{5}	427	434
MORZID	4.20 / 3.99	694	490	3.6	1.6	2.98×10^{6}	6.58×10^{4}	193	306
FIJYED	4.74 / 3.99	672	366	4.3	2.4	2.48×10^{6}	6.08×10^{4}	156	152
QOLVET	4.19 / 3.99	693	485	5.1	3.2	2.97×10^{6}	6.25×10^{4}	137	151
QIWDOR	4.48 / 4.05	98	92	1.5	1.1	3.00×10^{6}	6.79×10^{5}	66	83
SEHVOQ	5.95 / 3.89	345	301	6.2	3.9	2.36×10^{6}	3.04×10^{5}	56	77
WAZCUX	5.01 / 4.11	325	270	5.8	4.0	1.28×10^{6}	1.24×10^{5}	56	68
QOKCID	4.30 / 3.82	98	94	1.8	1.4	1.32×10^{6}	3.92×10^{5}	55	67
VUHJAK	4.40 / 4.24	134	113	2.2	2.0	4.56×10^{5}	5.38×10^{4}	61	57
IWELIG**	4.57 / 4.44	137	121	2.4	2.4	2.66×10^{6}	4.45×10^{5}	58	50

*Since the CoRE Mof 2019 database contains 8 possible models of the same MISQIQ structure, the table shows averaged data for all MISQIQ structure models.

**Since the CoRE Mof 2019 database contains 2 possible models of the same IWELIG structure, the table shows averaged data for all IWELIG structure models.

References

1. Malbrunot, P.; Vidal, D.; Vermesse, J.; Chahine, R.; Bose, T.K. Adsorbent Helium Density Measurement and Its Effect on Adsorption Isotherms at High Pressure. *Langmuir* **1997**, *13*, 539–544, doi:10.1021/la950969e.
2. Khoramzadeh, E.; Mofarahi, M.; Lee, C.-H. Equilibrium Adsorption Study of CO2 and N2 on Synthesized Zeolites 13X, 4A, 5A, and Beta. *J. Chem. Eng. Data* **2019**, *64*, 5648–5664, doi:10.1021/acs.jced.9b00690.
3. Sava Gallis, D.F.; Parkes, M.V.; Greathouse, J.A.; Zhang, X.; Nenoff, T.M. Enhanced O2 Selectivity versus N2 by Partial Metal Substitution in Cu-BTC. *Chem. Mater.* **2015**, *27*, 2018–2025, doi:10.1021/cm5042293.
4. Chowdhury, P.; Bikkina, C.; Meister, D.; Dreisbach, F.; Gumma, S. Comparison of Adsorption Isotherms on Cu-BTC Metal Organic Frameworks Synthesized from Different Routes. *Microporous Mesoporous Mater.* **2009**, *117*, 406–413, doi:10.1016/j.micromeso.2008.07.029.
5. Saha, D.; Bao, Z.; Jia, F.; Deng, S. Adsorption of CO2, CH4 , N2 O, and N2 on MOF-5, MOF-177, and Zeolite 5A. *Environ. Sci. Technol.* **2010**, *44*, 1820–1826, doi:10.1021/es9032309.
6. Kloutse, F.A.; Hourri, A.; Natarajan, S.; Benard, P.; Chahine, R. Experimental Benchmark Data of CH4, CO2 and N2 Binary and Ternary Mixtures Adsorption on MOF-5. *Sep. Purif. Technol.* **2018**, *197*, 228–236, doi:10.1016/j.seppur.2018.01.013.
7. Li, T.; Chen, D.-L.; Sullivan, J.E.; Kozlowski, M.T.; Johnson, J.K.; Rosi, N.L. Systematic Modulation and Enhancement of CO2 : N2 Selectivity and Water Stability in an Isoreticular Series of Bio-MOF-11 Analogues. *Chem. Sci.* **2013**, *4*, 1746, doi:10.1039/c3sc22207a.
8. Cmarik, G.E.; Kim, M.; Cohen, S.M.; Walton, K.S. Tuning the Adsorption Properties of UiO-66 via Ligand Functionalization. *Langmuir* **2012**, *28*, 15606–15613, doi:10.1021/la3035352.
9. Dubskikh, V.A.; Lysova, A.A.; Samsonenko, D.G.; Lavrov, A.N.; Kovalenko, K.A.; Dybtsev, D.N.; Fedin, V.P. 3D Metal–Organic Frameworks Based on Co(II) and Bithiophendicarboxylate: Synthesis, Crystal Structures, Gas Adsorption, and Magnetic Properties. *Molecules* **2021**, *26*, 1269, doi:10.3390/molecules26051269.
10. Pham, T.D.; Xiong, R.; Sandler, S.I.; Lobo, R.F. Experimental and Computational Studies on the Adsorption of CO2 and N2 on Pure Silica Zeolites. *Microporous Mesoporous Mater.* **2014**, *185*, 157–166, doi:10.1016/j.micromeso.2013.10.030.
11. Predescu, L.; Tezel, F.H.; Chopra, S. Adsorption of Nitrogen, Methane, Carbon Monoxide, and Their Binary Mixtures on Aluminophosphate Molecular Sieves. *Adsorption* **1997**, *3*, 7–25, doi:10.1007/BF01133003.
12. Li, S.; Chen, J.; Wang, Y.; Li, K.; Li, K.; Guo, W.; Zhang, X.; Liu, J.; Tang, X.; Yang, J.; et al. Adsorption and Separation of CH4/N2 by Electrically Neutral Skeleton AlPO Molecular Sieves. *Sep. Purif. Technol.* **2022**, *286*, 120497, doi:10.1016/j.seppur.2022.120497.
13. Cao, F.; Zhang, C.; Xiao, Y.; Huang, H.; Zhang, W.; Liu, D.; Zhong, C.; Yang, Q.; Yang, Z.; Lu, X. Helium Recovery by a Cu-BTC Metal–Organic-Framework Membrane. *Ind. Eng. Chem. Res.* **2012**, *51*, 11274–11278, doi:10.1021/ie301445p.
14. Zhao, Z.; Ma, X.; Li, Z.; Lin, Y.S. Synthesis, Characterization and Gas Transport Properties of MOF-5 Membranes. *J. Membr. Sci.* **2011**, *382*, 82–90, doi:10.1016/j.memsci.2011.07.048.
15. Zhang, Y.; Gao, Q.; Lin, Z.; Zhang, T.; Xu, J.; Tan, Y.; Tian, W.; Jiang, L. Constructing Free Standing Metal Organic Framework MIL-53 Membrane Based on Anodized Aluminum Oxide Precursor. *Sci. Rep.* **2015**, *4*, 4947, doi:10.1038/srep04947.
16. Kasik, A.; Dong, X.; Lin, Y.S. Synthesis and Stability of Zeolitic Imidazolate Framework-68 Membranes. *Microporous Mesoporous Mater.* **2015**, *204*, 99–105, doi:10.1016/j.micromeso.2014.10.050.
17. Hara, N.; Yoshimune, M.; Negishi, H.; Haraya, K.; Hara, S.; Yamaguchi, T. Diffusive Separation of Propylene/Propane with ZIF-8 Membranes. *J. Membr. Sci.* **2014**, *450*, 215–223, doi:10.1016/j.memsci.2013.09.012.
18. Yoo, Y.; Varela-Guerrero, V.; Jeong, H.-K. Isoreticular Metal−Organic Frameworks and Their Membranes with Enhanced Crack Resistance and Moisture Stability by Surfactant-Assisted Drying. *Langmuir* **2011**, *27*, 2652–2657, doi:10.1021/la104775d.
19. Tomita, T.; Nakayama, K.; Sakai, H. Gas Separation Characteristics of DDR Type Zeolite Membrane. *Microporous Mesoporous Mater.* **2004**, *68*, 71–75, doi:10.1016/j.micromeso.2003.11.016.
20. Liu, Y.; Zhang, B.; Liu, D.; Sheng, P.; Lai, Z. Fabrication and Molecular Transport Studies of Highly C-Oriented AFI Membranes. *J. Membr. Sci.* **2017**, *528*, 46–54, doi:10.1016/j.memsci.2017.01.012.
21. Nishiyama, N.; Ueyama, K.; Matsukata, M. Gas Permeation through Zeolite-Alumina Composite Membranes. *AIChE J.* **1997**, *43*, 2724–2730, doi:10.1002/aic.690431318.
